# Anatabine, Nornicotine, and Anabasine Reduce Weight Gain and Body Fat through Decreases in Food Intake and Increases in Physical Activity

**DOI:** 10.3390/jcm11030481

**Published:** 2022-01-18

**Authors:** Patricia E. Grebenstein, Paige Erickson, Martha Grace, Catherine M. Kotz

**Affiliations:** 1 Geriatric Research Education and Clinical Center, Minneapolis VA Medical Center, Minneapolis, MN 55417, USA; eric2920@umn.edu (P.E.); gracemkl@aol.com (M.G.); kotzx004@umn.edu (C.M.K.); 2 Food Science and Nutrition, University of Minnesota, St Paul, MN 55108, USA; 3 Integrative Biology and Physiology, University of Minnesota, Minneapolis, MN 55455, USA

**Keywords:** minor tobacco alkaloids, nicotine, body weight, obesity

## Abstract

Obesity is a leading cause of preventable death in the United States. Currently approved pharmacotherapies for the treatment of obesity are associated with rebound weight gain, negative side effects, and the potential for abuse. There is a need for new treatments with fewer side effects. Minor tobacco alkaloids (MTAs) are potential candidates for novel obesity pharmacotherapies. These alkaloids are structurally related to nicotine, which can help reduce body weight, but without the same addictive potential. The purpose of the current study was to examine the effects of three MTAs (nornicotine, anatabine, and anabasine) and nicotine on weight gain, body composition, chow intake, and physical activity. We hypothesized that the MTAs and nicotine would reduce weight gain through reductions in chow intake and increases in physical activity. To test this, male Sprague Dawley rats were housed in metabolic phenotyping chambers. Following acclimation to these chambers and to (subcutaneous (sc)) injections of saline, animals received daily injections (sc) of nornicotine, anabasine, anatabine, or nicotine for one week. Compared to saline-injected animals that gained body weight and body fat during the treatment phase, injections of nornicotine and anatabine prevented additional weight gain, alongside reductions in body fat. Rats receiving anabasine and nicotine gained body weight at a slower rate relative to rats receiving saline injections, and body fat remained unchanged. All compounds reduced the intake of chow pellets. Nornicotine and nicotine produced consistent increases in physical activity 6 h post-injection, whereas anabasine’s and anatabine’s effects on physical activity were more transient. These results show that short-term, daily administration of nornicotine, anabasine, and anatabine has positive effects on weight loss, through reductions in body fat and food intake and increases in physical activity. Together, these findings suggest that MTAs are worthy of further investigations as anti-obesity pharmacotherapies.

## 1. Introduction

Obesity is a leading cause of preventable death in the United States. It is characterized by an excess of adipose tissue and is positively correlated with the development of various diseases, including coronary heart disease, type II diabetes, stroke, cancer, and metabolic syndrome. According to the Center for Disease Control and Prevention, obesity affects over 40% of all adults and just under 20% of all children and adolescents in the United States, and accounts for just under $175 billion in annual spending in the United States [1].

There has been an increasing demand for more natural weight loss therapies [2], with over 30% of individuals attempting to jump start or increase their overall weight loss turning to non-prescription, over-the-counter supplement use [3]. This rise in interest in complementary and alternative medicine stems largely from the dissatisfaction with current long-term pharmacotherapy success, negative side effects, and the desire for quick and easy results [3]. Within the last 40 years, the US Food and Drug Administration (FDA) and/or the European Medicines Agency (EMA) have approved 9 anti-obesity drugs for long-term use [4]. While these pharmacotherapies are successful in treating obesity in the short term, many of these marketed drugs have been associated with rebound weight gain, negative side effects (particularly related to cardiovascular health), and the potential for drug abuse [4]. The negative side effects associated with these compounds and individual variability in responsiveness to these drugs underscore the need for additional pharmacotherapies for obesity with more desirable side effect profiles. 

Minor tobacco alkaloids are naturally occurring compounds [5] that are structurally [6] and functionally [7,8,9] analogous to nicotine, the primary psychoactive component in cigarettes, and have been the subject of research on novel therapeutics for smoking cessation [10], cognitive disorders including Alzheimer’s disease [11,12,13] and Parkinson’s disease [14], traumatic brain injury [15], inflammatory bowel disease [16] and psoriasis (Rock Creek Pharmaceuticals), as well as a novel strategy for pain management [17]. Previous research investigating the role of nicotine on body weight has repeatedly demonstrated that cigarette smoking and/or nicotine administration produces sustained weight loss and reduced food intake [18,19,20,21,22]. The similarities between the alkaloids and nicotine indicate there may be potential for the MTAs to reduce body weight, making them prime candidates for novel obesity pharmacotherapies. The MTAs include cotinine, nornicotine, anabasine, anatabine, and myosmine. These compounds are found in the tobacco plant [5], as well as in vegetables such as green tomatoes, red peppers, and potatoes [8], and as added ingredients to tobacco products at very low doses (between 8.64 and 1390 ug/g; [23]). The MTAs act at nicotinic acetylcholine receptors (nAChRs) [24,25,26,27], which are widely distributed throughout the central and peripheral nervous systems, and are involved in modulating the release of numerous neurotransmitters involved in reward and motivation [28,29,30]. The MTAs have more favorable pharmacokinetic profiles and lower relative potencies compared to nicotine [8,31], are less toxic and have a better safety index than nicotine [17].

The risk of abuse potential in humans for these compounds is unlikely. Anatabine has no reinforcing effects; it is not self-administered in rodents and is only a partial substitute for nicotine in drug-discrimination tasks [8,9]. In contrast, nornicotine is self-administered intravenously by rats [7], and can substitute for nicotine in drug-discrimination tasks with lower potency relative to nicotine. However, abuse of this compound on its own when delivered orally in low doses is unlikely considering the minimal reinforcement of nicotine replacement therapies. These nicotine replacement therapies, which deliver nicotine outside of the whole tobacco product environment, are delivered either orally, intranasally, or topically and have a much lower abuse potential [32]. Specifically, because of slower pharmacokinetics, and therefore more gradual increases in blood nicotine levels, little reinforcement from these products is obtained [33]. The same would be expected of the MTAs, which all have slower pharmacokinetics and reduced potencies relative to nicotine. Therefore, further investigations into these compounds are warranted.

Previous research on nicotinic acetylcholine receptor agonists on body weight initially focused on nicotine. Gradually, experimental studies [34,35,36,37,38] and review articles [39,40,41] have delved into the general role of the nicotinic acetylcholine receptor system in modulating energy balance. For example, an agonist at the alpha-7 nAChR subtype (TC-7020) has been shown to reduce weight gain and food intake in a mouse model of Type II Diabetes ([38]; for a comprehensive review of the role of α7 receptors on food intake behaviors, see McFadden et al. 2014). Similarly, the β2 agonist sazetidine-A and cytisine, a β4 agonist and partial β2 agonist, have also been shown to reduce food intake and body weight [26,42,43]. The MTAs, which interact with the nAChRs with varying potencies and binding affinities, represent a logical next step in understanding the role of nAChRs in modulating changes in energy balance and in the discovery of novel pharmacotherapies for the treatment of obesity.

We previously examined the dose–response relationship between nicotine and four MTAs (nornicotine, cotinine, anatabine, and anabasine) on food motivation and intake under food deprived conditions in 2 h operant sessions. All drugs, except for cotinine, produced reductions in food intake and motivation to respond for food pellets. These effects occurred within 30 min of injections and were able to produce sustained effects on food intake without rebounds in intake later in the day. These results suggested that, upon initial investigation and under very specific conditions, the MTAs could potentially reduce hunger and appetite for anti-obesity purposes. The purpose of the present study was to examine the effects of nornicotine, anabasine, and anatabine on weight gain, food intake, and physical activity across seven consecutive days of administration, with nicotine included as a positive control. We hypothesized that each of the MTAs, as well as nicotine, would reduce weight gain through reductions in food intake and increases in physical activity.

## 2. Materials and Methods

### 2.1. Animals

Forty-eight male Sprague Dawley rats (Charles River, Wilmington, MA, USA), initially 4 months old and weighing 300–350 g at arrival, were used in this study. This strain was chosen to extend our previous study examining changes in food intake during nicotine administration [44]. The age of animals was selected to allow for rats to reach maturity (>6 months old) prior to drug administration, to ensure that their growth curves had plateaued and drug administration would reduce diet-induced weight gain rather than prevent their natural growth. Upon arrival, all rats were individually housed in a temperature-controlled (21–22 °C) room in solid-bottom cages with corn-cob bedding and maintained on a 12 h light/dark cycle. Rodent chow (Harlan Teklad 8604) and water were allowed ad libitum. Protocols were approved by the Institutional Animal Care and Use Committee at the Minneapolis VA Health Care System in accordance with the 2013 NIH guide for the Care and Use of Mammals in Neuroscience and Behavioral Research.

### 2.2. Apparatus

At the start of the experiment, rats were housed individually in metabolic phenotyping chambers (Rat Promethion Continuous caging system; Sable Systems™, Las Vegas, NV, USA), in conditions like that of their home cage, with hanging food and water hoppers connected to inverted laboratory balances. Both food and water were available ad libitum. Animals were maintained on a standard 12 h light/dark cycle. Spontaneous physical activity was quantified via infrared beam breaks in three axes, X + Y + Z, and included locomotion, rearing, and grooming behaviors. Raw data were collected by SableScreen v2.2 (SableSystem ™) every second and extracted using Expedata v1.8.2 (SableSystem ™). Animals were housed within these chambers for 24 h/day. General housekeeping (refilling food hoppers and water bottles, weighing the animals, injections, etc.) occurred daily in the 1 h prior to the start of the dark cycle.

### 2.3. Drugs

The compounds (−)-nicotine bitartrate, (+/−)nornicotine, and (+/−)anabasine were obtained from Sigma Chemical Co. (St. Louis, MO, USA). The compound (+/−)anatabine was obtained from Toronto Research Chemicals, Inc. (North York, ON, Canada). All drugs were dissolved into sterile saline. The pH of all solutions was adjusted to 7.4 using dilute NaOH or HCl. All drugs were administered subcutaneously (sc) in a volume of 1 mL/kg. All drug doses are expressed as the base. The dose of each drug was as follows, nicotine: 0.50 mg/kg; nornicotine: 6.00 mg/kg; anatabine 3.00 mg/kg; anabasine 3.00 mg/kg. The doses selected were based on those used in our previous study evaluating the effects of the MTAs on deprivation-induced food intake [44]. The range of doses used in the previous investigation was narrowed down in the present study to a single dose of each drug that had previously produced reductions in food intake, without significant compensatory increases in food intake later in the day.

### 2.4. Experimental Procedure

#### 2.4.1. Acclimation

The experiment was run in three cohorts of sixteen. Rats were placed in the Sable Promethion chambers at the onset of the dark cycle and remained housed in these chambers for approximately 3 weeks. Acclimation to these chambers occurred for 5 days, during which food intake and body weights were manually recorded each day. Then, to acclimate animals to the injection procedure, all animals received daily injections of saline over four consecutive days (1 injection per day). Body weight and food intake continued to be manually recorded. Body composition was measured with EchoMRI™ at the end of the chamber acclimation period and again at the end of the saline injection period to obtain baseline measures of body composition and to ensure that repetitive injections did not have a negative impact on body composition (e.g., a reduction in lean mass).

#### 2.4.2. Treatment

Following acclimation to the chambers and injection procedure, the treatment phase began. Approximately 15 min prior to the onset of the dark cycle, animals were injected with a single dose of saline, nicotine, nornicotine, anatabine, or anabasine, for seven consecutive days (n = 8–10/group). Drug administration was counterbalanced between cohorts, such that each cohort of animals received all drugs and saline. Body weight and food intake were manually recorded each day during this period. The Sable Promethion chambers collected data on physical activity and food intake. At the end of the seven days, animals were evaluated for changes in body composition (EchoMRI™). Specifically, direct measurements of total body fat, lean mass, free water, and total body water were collected from live animals via ultrasound-based NMR. Animals were gently restrained in a clear plastic immobilization tube, placed into the EchoMRI chamber for analysis, and then returned to home cages.

### 2.5. Data Analysis

Body weight, body composition, food intake, and physical activity were the primary endpoint measures. Body weight was measured daily in the hour before lights off and was analyzed as cumulative weight gain (g) over time and as a percentage of the body weight on the final day of the baseline. Body composition was measured once per week in the two hours prior to lights off. Direct measurements of total body fat, lean mass, free water, and total body water were collected from live animals via ultrasound-based NMR (EchoMRI™, Houston, TX, USA), during a period of brief restraint (~3 min). Total body fat and lean mass were expressed as a percentage of lean and fat mass at the end of the saline injections. Food intake was measured daily; food was weighed manually in the hour before lights off and was analyzed as total cumulative food intake (g) over time and as a percentage of food intake on the final day of saline injections. Physical activity was measured within the Sable chambers. Physical activity, or distance traveled (i.e., mean meters traveled), was quantified via infrared beam breaks in three axes, X + Y + Z, and was expressed as a cumulative distance traveled and as a percentage of distance traveled on the final saline injection day. Physical activity was analyzed at several different timepoints: 24 h activity, which was then further broken down into the 12 h of the light and dark cycles and as absolute and cumulative activity at 6 h post-injection.

Parametric two-way analysis of variance (ANOVA) with Sidak multiple comparisons tests was performed to determine the role of time and drug type on each dependent measure, including weight gain, body composition, food intake, and physical activity. Statistical comparisons were both within and between subjects using repeated-measures analyses. Comparisons were made between the saline phase and subsequent days/weeks of treatment for within-subjects analyses, and between the different drugs throughout the various phases. Data were processed in Microsoft Excel. Statistical analyses and graphing were carried out using GraphPad Prism 9. All data are expressed as the mean values ± S.E.M.

## 3. Results

### 3.1. Body Weight

**Figure 1:**
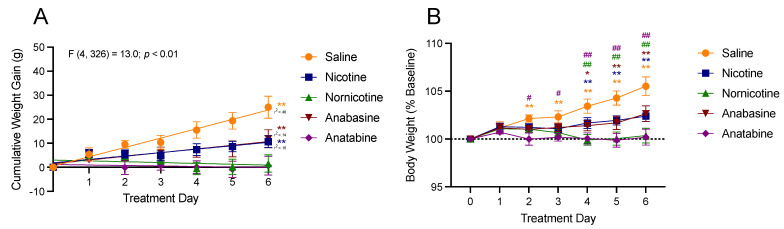
Mean (±S.E.M.) change in body weight over six days, following injections of either saline (orange; n = 8), nicotine (blue; n = 10), nornicotine (green; n = 10), anabasine (red; n = 10), or anatabine (purple; n = 10). Weight change is expressed relative to the mean body weight during the final day of the baseline phase (day 0) as absolute cumulative weight gain in grams (panel **A**) and as a percent change in body weight (panel **B**). * *p* < 0.05, ** *p* < 0.01 difference from baseline (day 0) body weight at the indicated day. # *p* < 0.05, ## *p* < 0.01 difference from weight gain in the saline group at the indicated day. Significance marks are color-coded to reflect which drug group experienced significant changes.

Figure 1 shows the effects of saline, nicotine, nornicotine, anatabine, and anabasine on weight gain during the 7 days of treatment, expressed as the cumulative weight gain relative to the final day of saline injections (Figure 1A) and as a percentage of the final day of saline injections (Figure 1B). There was a significant difference in the rate of weight gain across the seven days of treatment between the treatment groups (F (4, 326) = 13.00; *p* < 0.01). The slopes for saline (F (1, 54) = 50.74; *p* < 0.01), nicotine (F (1, 68) = 15.06), and anabasine (F (1, 68) = 10.72; *p* < 0.01) were all greater than zero, indicating some degree of weight gain during the seven days of treatment injections. To this end, weight gain was significantly different from baseline on days 2–6 in the saline group, on days 4–6 in the nicotine and anabasine groups. In contrast, animals that received daily injections of nornicotine and anatabine did not gain weight relative to the final day of saline injections, as demonstrated by a slope that was not significantly non-zero (nornicotine (F (1, 68) = 0.66; ns); anatabine (F (1, 68) = 0.20; ns)). There was also a significant main effect of drug type (F (4, 43) = 5.35; *p* < 0.01) and day (F (2.061, 88.63) = 15.00; *p* < 0.01) on cumulative weight gain, with a significant drug by day interaction (F (24, 258) = 4.52; *p* < 0.01). Post hoc tests revealed that when the data from rats receiving the drug treatments were compared to that of the rats receiving saline, weight gain was significantly less following injections of anatabine and nornicotine on days 2–6 and 4–6, respectively, without any significant differences from saline for nicotine and anabasine.

When the changes in body weight were transformed into a percentage of the final saline injection day, these differences were further clarified. As compared to the final day of saline injections (day 0 of drug treatment), there was also a significant main effect of drug type (F (4, 43) = 5.67; *p* < 0.01) and day (F (6, 258) = 16.06; *p* < 0.01), with a significant drug by day interaction (F (24, 258) = 4.72; *p* < 0.01) for weight gain. Specifically, there was a significant difference from baseline for saline on days 2–6, and nicotine and anabasine on days 4–6, but no significant difference from saline injections for nornicotine or anatabine on any day, indicating that nicotine and anabasine delayed weight gain, whereas nornicotine and anatabine prevented any weight gain. When the data from rats receiving the drug treatments were compared to that of the rats receiving saline, weight gain was significantly reduced following nornicotine administration on days 4–6 and anatabine administration on days 2–6.

### 3.2. Body Composition

**Figure 2:**
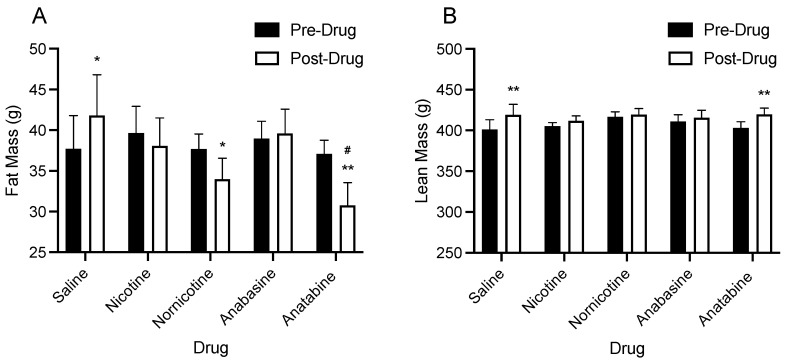
Mean (±S.E.M.) change in body composition before and after drug treatment, on the final day of saline injections (day 0; pre-drug) and the final day of the treatment phase (day 7; post-drug) in animals that received daily injections of either saline (n = 8), nicotine (n = 10), nornicotine (n = 10), anabasine (n = 10), or anatabine (n = 10) for 6 consecutive days. Body composition change is expressed as the contributions of fat mass (g; panel **A**) and lean mass (g; panel **B**) to overall body weight, as measured by EchoMRI. * *p* < 0.05, ** *p* < 0.01 difference from pre-drug to post-drug. # *p* < 0.05 difference from the saline group at the indicated timepoint.

Figure 2 shows the changes in body fat mass (Figure 2A) and lean mass (Figure 2B) before and after drug administration, on the final day of saline treatment (pre-drug) and the final day of drug treatment (post-drug; day 7) for saline, nicotine, nornicotine, anatabine, and anabasine. There was a significant main effect of timepoint (F (1, 43)–4.90; *p* < 0.01), but not drug (F (4, 43) = 0.75; ns), and a significant timepoint by drug interaction (F4, 43)–7.81; *p* < 0.01) for body fat mass. Post hoc comparisons revealed that relative to pre-drug body composition, animals receiving saline injections continued to gain weight (*p* < 0.05), which is in line with the continued growth expected from these animals. In contrast, nornicotine and anatabine administration significantly reduced fat mass after 6 days of administration (*p* < 0.01). Finally, there was no change in fat mass in animals that received daily injections of nicotine and anabasine. This suggests that these compounds delayed the normal fat mass gain that was observed in the rats within the saline group. This is in line with the body weight results, which showed that animals receiving nicotine and anabasine did not gain any additional weight during the treatment phase.

There was a significant main effect of timepoint (F (1, 43)–25.56; *p* < 0.01), but not drug (F (4, 43) = 0.22; ns), and a significant timepoint by drug interaction (F4, 43)–2.60; *p* < 0.05) for lean mass. Post hoc comparisons revealed that relative to pre-drug body composition, saline and anatabine administration produced significant increases in lean body mass (*p* < 0.01). In contrast, there was no change in lean mass after treatment with nicotine, nornicotine, or anabasine. There was no significant difference in lean mass at either timepoint for any drug relative to saline. These results suggest that after a week of treatment, nicotine and the MTAs do not significantly reduce lean mass.

### 3.3. Food Intake

**Figure 3:**
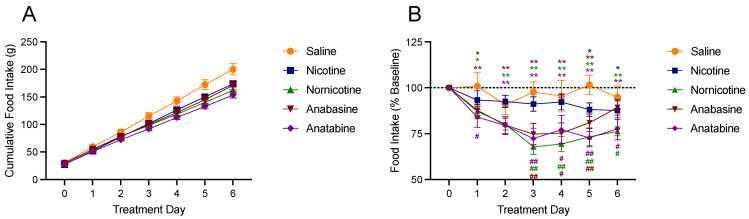
Mean (±S.E.M.) of changes in food (chow) intake over six days, following injections of either saline (orange; n = 8), nicotine (blue; n = 10), nornicotine (green; n = 10), anabasine (red; n = 10), or anatabine (purple; n = 10). Changes in food intake are expressed relative to the mean food intake during the final day of the baseline phase (day 0), as absolute cumulative change (panel **A**) and as a percent change (panel **B**) in food intake. * *p* < 0.05, ** *p* < 0.01 difference from baseline food intake (day 0) at the indicated day. # *p* < 0.05, ## *p* < 0.01 difference from food intake in the saline group at the indicated day. Significance marks are color-coded to reflect which drug group experienced significant changes.

Figure 3 shows the effects of saline, nicotine, nornicotine, anatabine, and anabasine on food intake during the first 6 days of treatment, expressed as the cumulative food intake relative to the final day of saline injections (Figure 3A) and as a percentage of the final day of saline injections (Figure 3B). Day 7 was not included in this analysis, because animals were removed from the chambers early to be placed in the body composition analyzer. There was a significant difference in the rate of cumulative intake across the treatment phase between the treatment groups (F (4,326) = 12.53; *p* < 0.01), indicating that all animals increased their food intake over the treatment phase. There was a significant main effect of drug (F (4,43) = 4.82; *p* < 0.01), day (F (6,258) = 2372.00; *p* < 0.01), and a significant drug by day interaction (F (24, 258) = 6.81; *p* < 0.01) on cumulative food intake across the 6 days of treatment. Specifically, when the data from rats receiving the drug treatments were compared to that of the rats receiving saline, cumulative food intake was significantly less for all drug treatment groups on days 4–6 and on day 3 for nornicotine and anatabine. Relative to the final day of saline injections, there was a significant increase in food intake for all days in all groups.

When the data from changes in food intake were transformed into a percentage of the final saline injection day, these differences were further extrapolated. When compared to the final day of saline injection (day 0), there was a main effect of drug type (F (4, 43) = 4.31; *p* < 0.01) and day (F (6, 258) = 22.90; *p* < 0.01), with a significant interaction (F (24, 258) = 2.62; *p* < 0.01) for food intake. Specifically, animals that received injections of saline continued to eat similar amounts of food relative to day 0. In contrast, there was a significant reduction in food intake from day 0 for nicotine on days 5–6, anabasine on days 1–5, and nornicotine and anatabine on all days. Similarly, when the data from rats receiving the drug treatments were compared to that of the rats receiving saline, weight gain was significantly reduced following anatabine on days 1 and 3–6, following anabasine on days 3–5, and nornicotine injections on days 3–6.

### 3.4. Physical Activity

**Figure 4:**
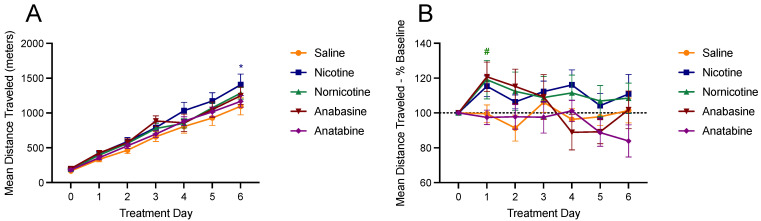
Mean (±S.E.M.) of changes in physical activity over six days, measured as the mean distance traveled in meters, following injections of saline (orange; n = 8), nicotine (blue; n = 10), nornicotine (green; n = 10), anabasine (red; n = 10), or anatabine (purple; n = 10). Changes in physical activity are expressed relative to the mean activity during the final day of the baseline phase (day 0), as absolute cumulative change (panel **A**) and as a percent change (panel **B**) in activity. * *p* < 0.05 difference from the saline group on the indicated day; # *p* < 0.05 difference from baseline physical activity (day 0) at the indicated day. Significance marks are color- coded to reflect which drug group experienced significant changes.

Figure 4 shows changes in physical activity, which are expressed relative to the mean activity during the final day of the baseline phase (day 0), as absolute cumulative change (Figure 4A) and as a percent change (Figure 1B) in activity. When analyzed cumulatively, there was a significant difference in SPA following injections of nicotine on day 6 (*p* < 0.05). There were no significant differences in cumulative SPA with the other drugs tested (F (4, 43) = 0.67; *ns*). When total SPA was analyzed across the entire day as a percent of the final saline injection day, there was a significant increase in mean SPA on day 1 for nornicotine (*p* < 0.05). There were no other significant differences with the other drugs tested (F (4, 42) = 1.42; *ns*).

**Figure 5:**
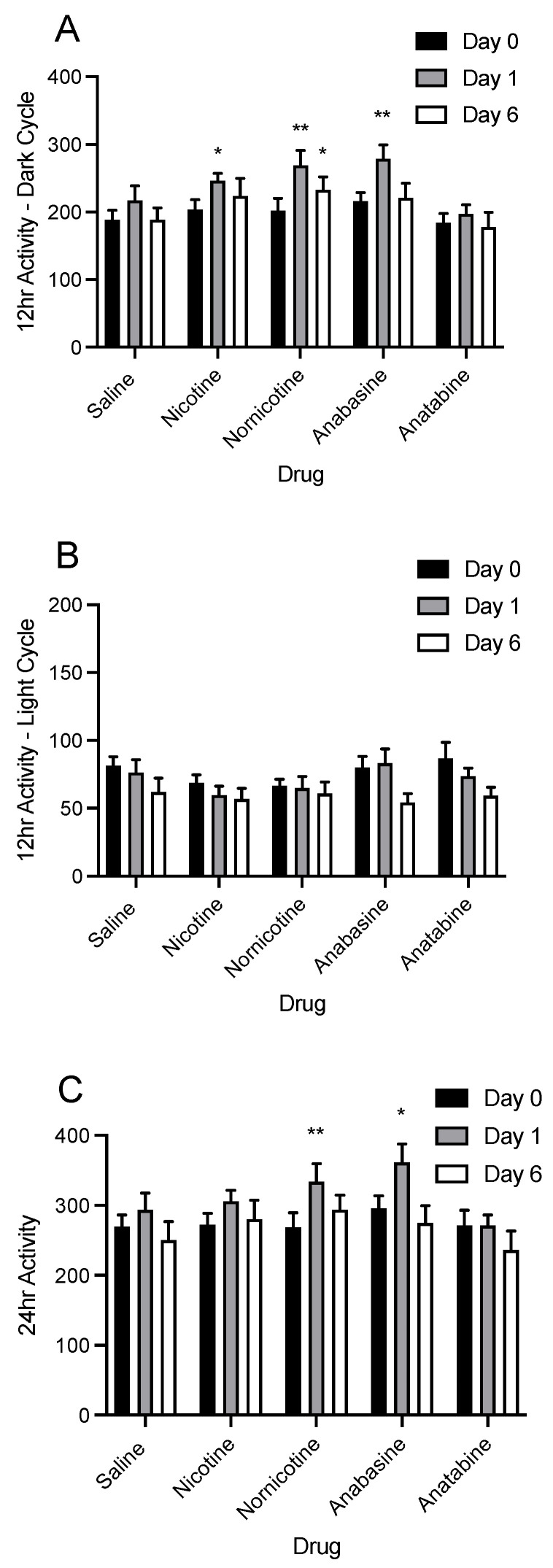
Mean (±S.E.M.) of changes in physical activity during the dark (panel **A**) and light (panel **B**) cycles, as well as the full 24 h period (panel **C**), for the final day of baseline (day 0), and days 1 of 6 of treatment injections of saline (n = 8), nicotine (n = 10), nornicotine (n = 10), anabasine (n = 10), or anatabine (n = 10). * *p* < 0.05, ** *p* < 0.01 difference from baseline activity (day 0) at the indicated day.

When data for SPA were broken down into the light and dark phases, there were significant changes in short-term SPA, as shown in Figure 5. While there was no main effect of drug on SPA (F (4, 43) = 2.56; *ns*), there was a significant increase in SPA on day 1, in the 12 h immediately following drug injection during the dark cycle, for injections of nicotine (*p* < 0.05), nornicotine (*p* < 0.01), and anabasine (*p* < 0.01; Figure 5A). Nornicotine produced sustained increases in total SPA throughout the treatment period (*p* < 0.05), such that dark cycle SPA was still increased on day 6 relative to baseline, whereas increases in SPA produced by the other drug treatments were no longer significant. Despite short-term effects of some of the alkaloids on SPA in the dark cycle, these effects did not extend to the light cycle (Figure 5B; (F (4, 43) = 0.93; *ns*). When the dark and light cycles were combined, there was an increase in 24 h SPA (Figure 5C) on day 1 for nornicotine (*p* < 0.01) and anabasine (*p* < 0.05).

**Figure 6:**
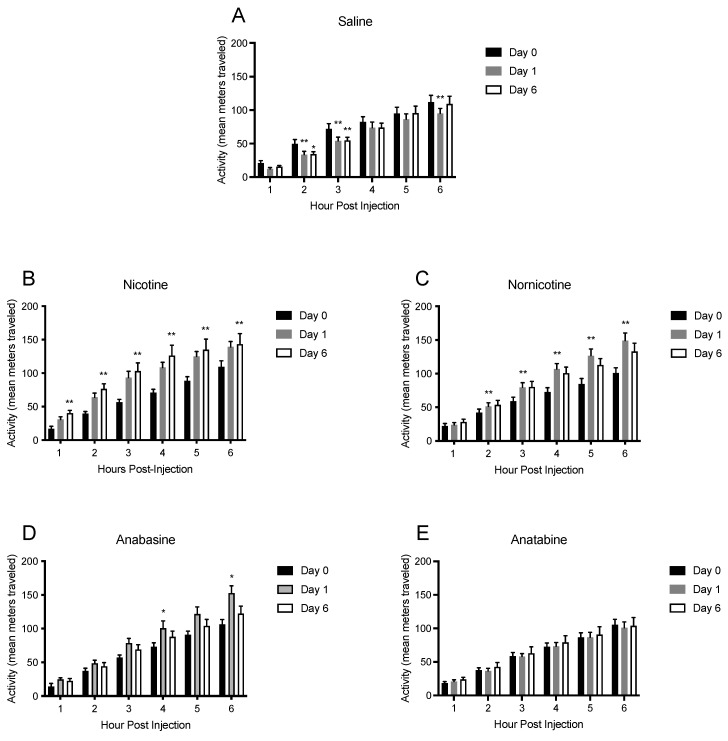
Mean (±S.E.M.) of cumulative changes in physical activity, measured as the mean distance traveled in meters during the 6 h following injections of saline (panel **A**; n = 8), nicotine (panel **B**; n = 10), nornicotine (panel **C**; n = 10), anabasine (panel **D**; n = 10), or anatabine (panel **E**; n = 10) for the final day of baseline (day 0), and days 1 and 6 of treatment injections. * *p* < 0.05, ** *p* < 0.01 difference from baseline activity (day 0) at the indicated hour.

**Figure 7:**
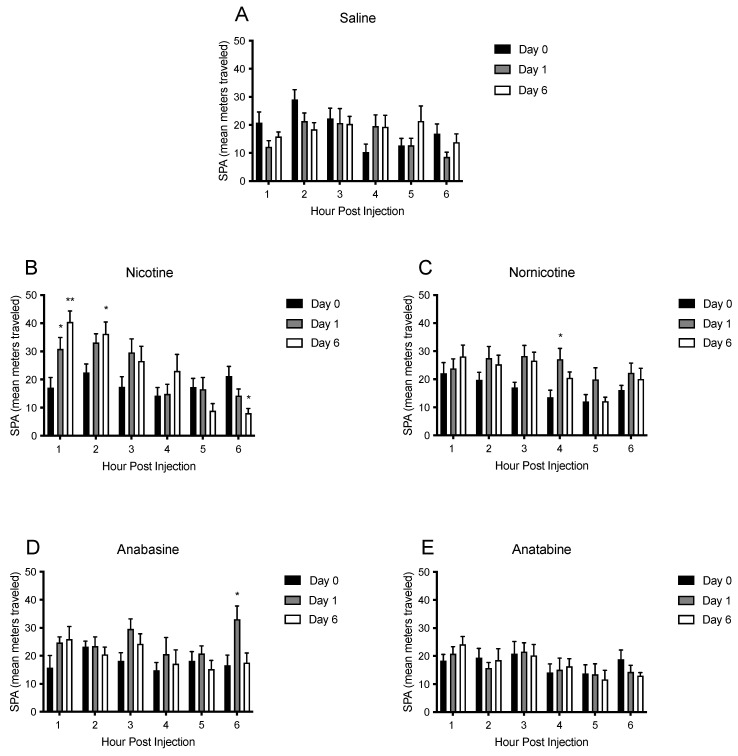
Mean (±S.E.M.) of absolute changes in physical activity, measured as the mean distance traveled in meters during the 6 h following injections of saline (panel **A**; n = 8), nicotine (panel **B**; n = 10), nornicotine (panel **C**; n = 10), anabasine (panel **D**; n = 10), or anatabine (panel **E**; n = 10) for the final day of baseline (day 0), and days 1 and 6 of treatment injections. * *p* < 0.05, ** *p* < 0.01 difference from baseline activity (day 0) at the indicated hour.

To better understand the time course of the effects of nicotine and the MTAs on SPA, hourly changes in SPA were also analyzed over the first 6 h post-injection. These analyses revealed changes in short-term SPA, as shown in Figure 6 and Figure 7 and Supplementary Materials, Figures S1 and S2. Total SPA was analyzed every hour for the first 6 h on the final day of saline injections and throughout all days of the treatment phase. For clarity, these data are summarized in Figure 6 and Figure 7 and are presented as changes in SPA within the first 6 h of baseline, day 1, and day 6 both as cumulative SPA (Figure 6) and hourly SPA (Figure 7).

Within the saline group (Figure 6A), there was a significant main effect of hour (F (5,35) = 109.4; *p* < 0.01), but not treatment day (F (2, 14) = 3.23; *p* = 0.07), on cumulative SPA, which indicated an increase in total cumulative SPA over time, as expected (Figure 6A). There were significant, transient reductions in SPA in hour 3 on days 1, 2, and 7 relative to saline injections (Figure S1A). In general, however, SPA in saline-treated animals remained consistent within the first six hours following injection. Data were also analyzed for hourly changes in SPA, as opposed to cumulative SPA (Figure 7A). Again, there was a significant main effect of hour (F (5, 35) = 4.47; *p* < 0.01), but not treatment day (F (2, 14) = 0.28; *ns*), indicating stability among SPA in the saline group, although a reduction in SPA was seen in hour two on days 6 and 7 and in hour 4 on day 7 (Figure S2A).

Within the nicotine treatment group, there was a significant main effect of hour (F (5, 35) = 131.0; *p* < 0.01), and treatment day (F (2, 18) = 9.32; *p* < 0.01) on SPA in the six hours following injection (Figure 6B). Specifically, SPA was increased in the second hour for all days, except day 1 (Figure S1B) and was maintained in all subsequent days for hours 3–6. When analyzed for hourly changes (Figure 7B), there was a main effect of hour (F (5, 45) = 8.62; *p* < 0.01), but not treatment day (F (2, 18) = 2.69; *p* < 0.05), with a significant interaction between hour and treatment day (F (10, 90) = 5.81; *p* < 0.01). Specifically, nicotine produced increases in SPA in hour one on all treatment days, relative to baseline, in hour two on days 2–7, and in hour 3 on days 1 and 7 (Figure S2B). These results suggest that nicotine increased SPA within the first 2–3 h following administration.

Nornicotine administration produced significant increases in SPA as well. There was a significant main effect of hour (F (5, 45) = 199.80; *p* < 0.01), and treatment day (F (2, 18) = 13.97; *p* < 0.01), and a significant hour by treatment day interaction (F (10, 90) = 14.43; *p* < 0.01) on cumulative SPA (Figure 6C). Spontaneous physical activity was increased following nornicotine injections in hour 2 on days 2, 4, and 6, and in hours 3–6 on all days (Figure S1C). When analyzed hourly (Figure 7C), there was a main effect of hour on SPA (F (5, 45) = 5.58; *p* < 0.01), and on treatment day (F (2, 18) = 16.68; *p* < 0.01), without an interaction between hour and treatment day (F (10, 90) = 0.89; *ns*). Post hoc tests revealed significant hourly increases in SPA relative to baseline at just five timepoints (hour 3 on days 1 and 5, hour 4 on days 1 and 2, and hour 5 on day 7), indicating that nornicotine produced small increases in SPA in the hours following injection, which led to significant additive increases over time (Figure S2C).

Animals that received injections of anabasine showed less pronounced increases in SPA. There was a significant main effect of hour (F (5, 40) = 204.0 *p* < 0.01), and treatment day (F (2, 16) = 4.07; *p* < 0.05) with a significant hour by treatment day interaction (F (10, 80) = 3.08; *p* < 0.01) on cumulative SPA (Figure 6D). Specifically, SPA was increased following anabasine injections at hours 3 on day 1, hour 4 on days 1, 6, and 7, hour 5 on days 1 and 7, and hour 6 on days 1, 6 and 7 (Figure S1D). When SPA was analyzed hourly (Figure 7D), there was no main effect of treatment day (F (5, 45) = 1.14; *p* < 0.05), but there was an effect of hour (F (2, 18) = 10.60; *p* < 0.01) without a significant interaction (F (10, 90) = 1.40; *ns*). Significant hourly increases in SPA were seen in hour 4 on days 12 and 7, and in hour 6 on day 1 (Figure S2D). These results suggest that at the dose of anabasine tested, the drug is only able to produce transient and minimal effects on SPA.

Similar effects were seen following injections of anatabine. There was a significant main effect of hour (F (5, 45) = 124.1; *p* < 0.01), but not treatment day (F (2, 18) = 0.26; ns), and without an interaction (F (10, 90) = 0.30; ns) on cumulative SPA (Figure 6E). SPA was increased in hours 2 and 3 on days 3–4, in hour 4 on days 2–4, and in hour 5 on days 2 and 7 (Figure S1E). When SPA following anatabine injections was analyzed hourly (Figure 7E), there was a main effect of hour (F (5, 45) = 4.20; *p* < 0.01), but not treatment day (F (2, 18) = 0.08; *ns*), and no interaction (F (10, 90) = 0.52; *ns*). Post hoc analyses did not reveal any timepoints where SPA was increased (Figure S2E), although a decrease relative to baseline was seen in hour 6 on day 7. These results suggest that at the dose tested, anatabine produced short acting, minimal increases in SPA over time.

A comparison between saline, nicotine and the MTAs alkaloids for SPA within the first 6 h of day 1 of treatment (not depicted in a figure), saw significant main effects of drug type (F (4, 37) = 6.88; *p* < 0.01), and hour (F (5, 182) = 374.2; *p* < 0.01), with a significant interaction between drug type and hour (F (20, 182) = 3.83; *p* < 0.01). Post hoc comparisons revealed that nicotine produced the largest effects on SPA and anatabine the smallest effects. Nicotine-induced increases in SPA were greater than saline in hours 2–6 and greater than anatabine in hours 3–6. Nornicotine and anabasine were also greater than saline in hours 4–6, and greater than anatabine in hours 4–5.

## 4. Discussion

### 4.1. Body Weight, Body Composition, and Food Intake

The present study examined the effects of one week-long administration of nornicotine, anatabine, and anabasine, alongside nicotine, on food intake, body weight, body composition, and physical activity. The animals that received injections of nicotine and anabasine gained weight at a slower rate compared to the saline-treated animals, which gained weight over time. The rats injected with nornicotine and anatabine maintained their body weight between the baseline and treatment phases. Similarly, while saline-treated animals gained body fat over time, rats that received nicotine and anabasine did not gain body fat and those that received nornicotine and anatabine lost body fat after one week of injections. These results suggest that of the compounds tested, nornicotine and anatabine exert the strongest effects on body weight by causing a loss of body fat and a subsequent pause in weight gain.

Previous studies examining the effects of nicotine administration on body weight in rodents consistently demonstrated that nicotine prevents weight gain and/or produces weight loss [19,45,46,47,48,49,50,51,52,53,54]. Similarly, other studies have also shown that nicotine administration specifically reduces fat mass in rodents [46,51,53,54,55]. The present study expands on this work by examining the effects of MTAs on body weight and body composition, for which no previous research exists. The complete prevention of weight gain and reduction in body fat following administration of nornicotine and anatabine indicates a strong potential for these alkaloids to be used in the treatment of obesity.

The effects of the MTAs and nicotine on body weight and body composition were facilitated by changes in food intake and SPA. All MTAs tested, along with nicotine, reduced food intake. The greatest effects on food intake were seen following injections of nornicotine and anatabine, whereas anabasine produced more transient effects. Our previous investigation into the effects of the MTAs on food intake, which occurred during 2 h operant sessions under food-restricted conditions, found similar reductions in food intake with the MTAs [44]. The current study builds on that dose–response study, by removing the deprivation component and examining food intake following daily injections of a single drug and single dose for one week. At doses equivalent to those used in Bunney et al. (2018), nornicotine (6.00 mg/kg), anatabine (3.00 mg/kg) and anabasine (3.00 mg/kg) elicited more pronounced reductions in food intake compared to nicotine (0.50 mg/kg). The dose of nicotine used in these studies is significantly lower than that used in previous studies, where doses of nicotine were typically between 1–12 mg/kg/day with minipumps [48,49,50,56,57] and 2–4 mg/kg/day (multiple injections of 0.40–1.00 mg/kg per injection) with intraperitoneal (i.p.) injection [58,59], so the relatively modest decrease in food intake following injections of nicotine in our study is unsurprising.

Other than Bunney et al. 2018, to our knowledge, only one other study has examined changes in food intake following administration of the MTAs. Caine et al. 2014 examined the dose–response relationship between the MTAs and food-maintained responding and characterized the doses at which responding for food was reduced by 50% in mice [8]. For nornicotine, anatabine, and anabasine, those doses were 8.23, 22.8, and11.3 mg/kg, respectively. The doses administered in the present study to rats were considerably lower (3.0–6.0 mg/kg) but were still able to produce 20–30% reductions in food intake.

The ability of the MTAs and nicotine to reduce body weight and food intake is likely mediated by their effects on nicotinic acetylcholine receptors (nAChRs). Nicotine and the MTAs are agonists at nAChRs. These receptors are ligand-gated ion channels, comprised of five membrane-spanning subunits, with twelve α and β subunits, which combine into various subtypes [30]. The nAChRs are widely distributed throughout the central and peripheral nervous systems and are involved in modulating the release of numerous neurotransmitters, including dopamine, which likely account for their ability to mediate nicotine reinforcement and withdrawal [30]. The nAChRs are also located within the arcuate nucleus of the hypothalamus; an area responsible for regulating energy homeostasis through two sets of neurons: proopiomelanocortin (POMC)/cocaine and amphetamine regulated transport (CART) and neuropeptide-Y (NPY)/Agouti gene-related protein (AgRP) neurons, which interact to facilitate increases (via NPY/AgRP) or decreases (via POMC/CART) in food intake [60]). There is extensive evidence indicating that the arcuate nucleus is involved in mediating nicotine’s effects on body weight [61,62,63,64,65], and nAChRs have been identified on POMC neurons in the arcuate nucleus [26,66], specifically the α2, β4, and α7subunits [66]. Neuropeptide Y and POMC have also been shown to be involved in nicotine’s ability to reduce food intake during nicotine administration. For example, nicotine administration increases the firing rate of POMC neurons [67], whereas POMC knockout mice fail to show an effect of nicotine on food intake, compared to wild type mice [26]. It is likely that any effects the MTAs have on food intake, physical activity, and metabolism, are similarly regulated by neuropeptides within the arcuate nucleus.

While nicotine and the MTAs are all agonists at nAChRs, these compounds differ with respect to their selectivity, potency, and efficacy for the various nAChR subtypes [24]. Such differences in potencies between nicotine and the MTAs, with higher doses of MTAs needed to achieve similar behavioral effects, likely account for the varied effects seen within the present study. Potency differences have been previously shown in ours’ and others’ studies to contribute to alterations in food intake [44,68] and other behaviors, such as intracranial self-stimulation [69]. Nornicotine has been shown to be ~10-fold less potent, anatabine ~7-fold less potent, and anabasine ~4-fold less potent than nicotine [68] for reducing food maintained responding. Our previous study showed similar potency rankings, although we found anatabine to be more potent than anabasine with respect to reducing food intake under deprived conditions [44]. The differences in potencies between nicotine and the MTAs may be due to differences in their neural mechanisms of action [70], pharmacokinetics [71], or both. For example, differences in receptor binding between nicotine and the MTAs, as determined by the relative affinity for the MTAs at various nAChR subtypes (α4β2, α7, and α3β4), likely play a role in the differences in food intake seen within the present study and is summarized in Withey et al. (2018). Likewise, differences in the half-lives between nicotine and the MTAs, (ranging from 9 to 20 h; Jacob et al. (1999)), may also contribute to the differences in food intake throughout the present study. Although studies have shown that nicotine can produce conditioned taste aversions [72], this is unlikely to be the sole or primary cause of the reductions in food intake by nicotine and the alkaloids seen in our study. Considering the effects of nAChRs on food motivation, it is more likely that the reductions in food intake seen in the present study reflect a change in food motivation, as opposed to feelings of malaise. Future investigations into the MTAs as potential pharmacotherapies for obesity should determine the extent to which they may produce conditioned taste aversions. Furthermore, it will be important to examine their effects on more palatable foods, and in animal models of diet-induced obesity.

### 4.2. Physical Activity and Energy Expenditure

The effects of the MTAs on spontaneous physical activity (SPA) over 24 h were non-significant, but hourly analyses revealed significant changes in physical activity in the hours immediately following injections. Specifically, nicotine increased SPA in the 6 h post-injection, and nornicotine produced small increases in SPA over time that resulted in net increases in SPA overall. In comparison, anabasine and anatabine produced relatively minor increases in SPA that had a minimal effect on overall activity over 24 h. The time course of these effects is important for understanding what potential dosing frequency might be needed should these compounds be further pursued to treat obesity.

The increases in SPA seen following nicotine injections are somewhat in line with previous research. The behavioral sensitization effects of nicotine, which are typically seen as an escalation of increased locomotor activity over time with repeated drug administration, have been well documented (for a thorough review of this phenomenon, please refer to Mao and McGehee 2010 [73]). While we did observe increased SPA in animals that received daily injections of nicotine, we failed to see an enhancement of physical activity over time, as would be expected with behavioral sensitization. However, behavioral sensitization is not always observed in investigations on nicotine. For example, there are several studies that have documented a decrease in physical activity following an acute dose of nicotine within the first 10 min following administration [74]. We did not examine physical activity at such a fine resolution in the present study, and it is possible that there was a reduction in activity immediately following the injections of nicotine. This would coincide with the reduction in food intake seen in our previous investigation into the effects of nicotine and MTA administration on deprivation-induced food intake [44]. In that study, we observed a reduction in food intake immediately following administration of nicotine. Regardless of a lack of behavioral sensitization in the present study, we did still observe an increase in nicotine-induced physical activity, which has also been demonstrated in previously [75,76,77,78,79].

We were unable to be explicitly evaluate changes in energy expenditure in the present study, but it is likely that the increases seen in physical activity following nicotine injection led to increases in energy expenditure, which contributed to the weight loss and body fat effects seen in the present study. Numerous investigations have detailed the effects of nicotine on energy expenditure, many of which support the notion the energy expenditure is increased with nicotine administration in both animal models [77,80,81] and in humans [20,82,83,84,85,86]. While some studies have failed to find any effect of nicotine on energy expenditure, these studies have typically been limited in drug access, differed in route and schedule of drug administration, and/or been unable to continuously measure energy expenditure [48,52,55,87].

The effects of the MTAs on physical activity are not well characterized. Most MTA studies have focused on nornicotine, and its ability to increase physical activity has been demonstrated. Specifically, nornicotine has been shown to increase locomotor activity in rats at doses comparable to the present study (3 mg/kg in Wang 2020 [88]; 5 mg/kg in Green 2002 [89]). These increases in physical activity following nornicotine administration are accompanied by increases in dopamine synthesis and are blocked by D2 antagonists and nucleus accumbens lesions [89]. Investigations into the effects of anatabine on physical activity support the results from the present study demonstrating little to no increases in activity [90] or an initial reduction in activity [91] despite increases in dopamine release within the nucleus accumbens [91]. In a comparison study investigating the effects of nicotine and MTAs on locomotor activity in rats [92], nicotine, nornicotine, and anabasine all produced increases in activity following administration of at least one dose tested per drug, with the rank order of potency listed as (-)-nicotine > (+)-nornicotine > (+)-nicotine > cytisine > lobeline > anabasine, which would be comparable to the inferred potency rankings of the drugs tested in the present study (nicotine > nornicotine > anabasine > anatabine). It is possible that the increases in physical activity, although small, contributed to changes in body weight and body composition within the present study.

Nicotinic acetylcholine receptors are located within the peripheral nervous system [93,94], as well as the central nervous system, which suggests that the MTA and nicotine effects on body weight may be partially due to their effects on non-neuronal tissues (for a comprehensive review of the role of nicotinic cholinergic signaling in adipose tissue, please refer to Somm (2014) [95]). Numerous nAChR subtypes are found on white and brown adipose tissue in rodents [96] and humans [97], and on autonomic ganglia in control of adipose tissue activity [98]. Nicotine appears to facilitate the transition from fat storage in adipose tissues toward utilization of fat by the muscle [53], is associated with increased BAT thermogenesis [77], and produces increased levels of UCP-1 [53] and decreased levels of lipoprotein lipase activity [99]. Nicotinic acetylcholine receptors have also been implicated in anti-inflammatory pathways. Specifically, the cholinergic anti-inflammatory pathway [100] is activated following administration of nicotine (Wang et al. 2011), whereas genetic knockouts of the α7 nAChR subtype show increased pro-inflammatory cytokine production [101]. Recent research into the use of anatabine for treatment of chronic inflammatory disorders, including colitis [16], muscular sclerosis, Hasimoto’s thyroiditis, and psoriasis (Rock Creek Pharmaceuticals), and cognitive disorders including Alzheimer’s disease [12], highlight the ability of anatabine, and likely other MTAs to facilitate activation of anti-inflammatory pathways. Specifically, anatabine has been shown to suppress two key mediators of inflammation, NF-κB and STAT3 [12], thereby reducing inflammatory cytokine expression of TNF-α and others [16]. Furthermore, activation of peripheral nAChRs on pancreatic islet cells by nicotine (please see Somm 2014 [95] for review) reduces pro-inflammatory cytokines and increases levels of anti-inflammatory cytokine production within the pancreas in diabetic models [102]. While not studied in the context of obesity, it is likely that anatabine’s effects, and by extension nornicotine’s effects, on pro-inflammatory cytokines are at least partially responsible for its anti-obesogenic properties.

### 4.3. Future Directions

The future use of the minor tobacco alkaloids as therapeutics for obesity hinges on further elucidating their efficacy in more complex animal models of obesity and characterizing their potential toxicity. Specifically, the effects of these compounds in diet-induced obesity models will provide critical information regarding their effects on palatable food intake, changes in adiposity, and interactions with reward systems. In addition, further research exploring the potential toxic and negative side effects of these compounds is also warranted. For example, investigations into the anxiogenic effects of the MTAs are crucial steps for advancing these compounds as pharmacotherapies in humans. In addition, while our previous research indicates that gastrointestinal malaise is unlikely to be facilitating the reductions in food intake [44], conditioned taste aversion studies would help add further support to this finding. Lastly, complete toxicological evaluations of these compounds in humans will be necessary. At this point, the most thoroughly studied MTA in humans is anatabine. Its investigation in human clinical trials for the treatment of inflammatory disorders [102,103] and muscle strength recovery [104] has demonstrated that the compound is safe and well tolerated in humans at clinically relevant doses and has no measurable effects on heart rate or blood pressure. In addition, because anatabine and the other MTAs act as full or partial agonists with reduced potencies at the nAChRs, it is likely that these compounds are less toxic than nicotine. While little research has been performed on nornicotine and anabasine toxicity, particularly in humans, research in animal models [17,105] indicates reduced toxicity of nornicotine and anabasine relative to nicotine. These compounds have traditionally been investigated in the context of their relationship to nicotine, and future studies will need to evaluate their potential toxicity further.

## 5. Conclusions

All MTAs tested produced effects on body weight and body composition, with the most prominent effects seen with the administration of nornicotine and anatabine. While anabasine injections slowed body weight gain and prevented fat mass gain, nornicotine and anatabine reduced body weight and fat mass. These two compounds also had the greatest effects on food intake, whereas only nornicotine (and nicotine) produced sustained increases in physical activity. These results support the further investigation of nicotine and anatabine as potential pharmacotherapies for obesity.

## Data Availability

The data presented in this study are available on request from the corresponding author.

## Supplementary Materials

The following are available online at https://www.mdpi.com/article/10.3390/jcm11030481/s1, Figure S1: Mean (±SEM) of cumulative changes in physical activity, measured as the mean distance traveled in meters during the 6 h following injections of saline, nicotine, nornicotine, anabasine, or anatabine the final day of baseline, and days 1 through 6 of treatment injections. Figure S2: Mean (±SEM) of absolute changes in physical activity, measured as the mean distance traveled in meters during the 6 h following injections of saline, nicotine, nornicotine, anabasine, or anatabine for the final day of baseline, and days 1 through 6 of treatment injections.
