# Supplementary material for: Anatabine, Nornicotine, and Anabasine Reduce Weight Gain and Body Fat through Decreases in Food Intake and Increases in Physical Activity
Source: J Clin Med. 2022 Jan 18;11(3):481. doi: 10.3390/jcm11030481 (PMC8837150; doi:10.3390/jcm11030481)
Supplement: Supplementary file 1 [file jcm-11-00481-s001.zip › jcm-1502043-supplementary.pdf]

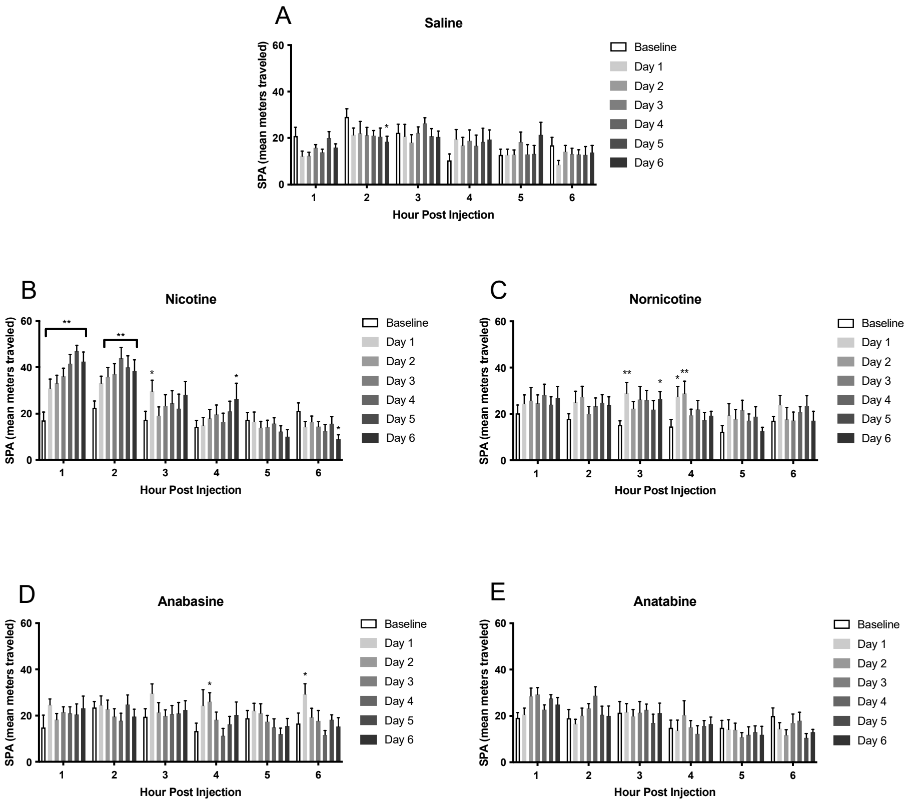

Figure S1. Mean (± SEM) of cumulative changes in physical activity, measured as the mean distance traveled in meters during the 6hrs following injections of saline (panel A; n=8), nicotine (panel B; n=10), nornicotine (panel C; n=10), anabasine (panel D; n=10), or anatabine (panel E; n=10) for the final day of baseline (day 0), and days 1 through 6 of treatment injections. *$p < 0.05$, **$p < 0.01$ difference from baseline activity (day 0) at the indicated hour for the indicated day.

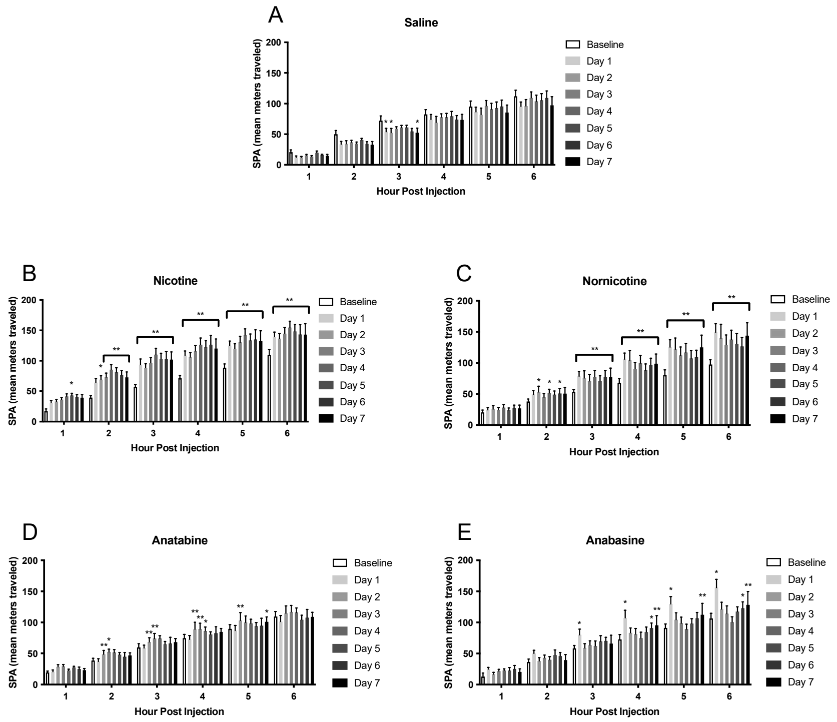

Figure S2. Mean (±SEM) of absolute changes in physical activity, measured as the mean distance traveled in meters during the 6 h following injections of saline (panel A; n = 8), nicotine (panel B; n = 10), nornicotine (panel C; n = 10), anabasine (panel D; n = 10), or anatabine (panel E; n= 10) for the final day of baseline (day 0), and days 1 through 6 of treatment injections. * $p < 0.05$, ** $p < 0.01$ difference from baseline activity (day 0) at the indicated hour on the indicated day.
